# Modeling Extreme Rainfall Using the Generalized Extreme Value Distribution and Exceedance Analysis in Colima, Mexico

**DOI:** 10.3390/s26020532

**Published:** 2026-01-13

**Authors:** Raúl Renteria, Raúl Aquino, Mayrén Polanco

**Affiliations:** 1 Facultad de Ingeniería Civil, Universidad de Colima, Coquimatlán 28400, Mexico; rrenteria5@ucol.mx; 2 Coordinación General de Investigación Científica, Universidad de Colima, Colima 28040, Mexico; 3 Facultad de Economía, Universidad de Colima, Villa de Álvarez 28970, Mexico; mayrenpg@ucol.mx

**Keywords:** GEV, exceedance analysis, sensors networks, extreme rainfall

## Abstract

This study develops a statistical and technological framework to analyze extreme rainfall in Colima, Mexico, by integrating historical precipitation records, probabilistic modeling, and spatial visualization. Using data from CONAGUA meteorological stations, we identify high-intensity rainfall events and model their recurrence using the Generalized Extreme Value (GEV) distribution to estimate key return periods. The results support flood-risk assessment and territorial planning in Colima. Spatial interpolation was performed in Python (version 3.13), and QGIS (version 3.38) produces exceedance maps that illustrate geographic variations in rainfall intensity across the state. These exceedance maps reveal a consistent spatial pattern, with the northern and western areas of Colima experiencing the highest frequencies of extreme events. Based on these results, the integration of real-time sensor technologies and satellite observations may improve flood monitoring and risk management frameworks.

## 1. Introduction

Extreme hydrometeorological events have become a major environmental challenge worldwide, with increasingly severe consequences for vulnerable populations and ecosystems. UN-Habitat reports that water- and climate-related problems account for approximately half of the human and economic damage caused by disasters over the last 50 years [1].

In Mexico, hydrometeorological events resulted in approximately USD 4.85 billion in losses in 2023, significantly impacting municipalities in the state of Colima [2]. These events disrupt infrastructure, livelihoods, and natural resources, increasing disaster risk and social vulnerability.

Climate change is now causing more frequent and severe disasters. These events create urgent threats that need rapid, innovative action. Between 2005 and 2015, about 1.5 billion people were affected by climate disasters. Many lives were lost, and massive displacement occurred, mainly because of hydrometeorological events [3]. From 2014 to 2023, UNDRR data show the crisis worsened. The number of people affected rose to about 2028 per 100,000 inhabitants—over 124 million each year. This highlights the growing danger and rising vulnerability worldwide [4].

Small Island Developing States (SIDS), Least Developed Countries (LDCs), and the most vulnerable populations face particularly high risks [4]. Greenhouse gas emissions significantly influence the magnitude and frequency of natural phenomena, especially affecting the hydrological cycle and rainfall patterns in Mexico [5,6].

Therefore, urgent action is needed to develop and implement climate change adaptation strategies for disaster management. The state of Colima, in western Mexico, exemplifies this situation due to its geographic location and orographic characteristics, which favor the occurrence of phenomena such as hurricanes, tropical storms, and torrential rains, primarily affecting the coastal area (Table 1).

The heavy rainfall associated with these events causes flooding and landslides, resulting in economic, environmental, and social consequences that impact regional development and compromise the safety of the population.

Understanding the statistical behavior of extreme rainfall is vital for effective risk management and land-use planning.

To address this need, traditional indicators such as averages or annual totals cannot capture the behavior of rare, high-impact rainfall events. Extreme value theory is better suited for this purpose, as it focuses on the statistical behavior of extremes. Within this framework, the generalized extreme value (GEV) distribution enables the estimation of return periods and understanding of the recurrence of severe hydrometeorological events linked to flood risk.

Integrating statistical methods with Geographic Information Systems (GIS) and cloud-based data processing platforms has proven effective for improving disaster prevention and response. In this study, extreme rainfall analysis relies on data from CONAGUA meteorological stations, which function as a distributed in situ environmental sensor network for precipitation monitoring in Colima.

Although these stations do not constitute a fully real-time Internet of Things (IoT) system, they provide the foundational sensing infrastructure for IoT-enabled monitoring architectures. In this context, the present work leverages historical sensor data to characterize extreme rainfall patterns, while future extensions may incorporate real-time IoT sensors, satellite-based observations, and statistical downscaling techniques to enhance spatial resolution and early warning capabilities for flood risk assessment in the state of Colima.

## 2. Materials and Methods

### 2.1. Field Camp

The study was conducted in the state of Colima, located on the central-western coast of the Mexican Pacific. Its topography ranges from coastal plains to mountainous areas associated with the Sierra Madre del Sur and the Colima Volcano, both of which directly influence the spatial distribution of precipitation. The climate is tropical, with a pronounced rainy season between June and October.

As a representative example for illustrating the exceedance analysis and extreme-value modeling procedures, meteorological station 6001, located in Armeria, Colima (latitude 18.93833333° N, longitude −103.9463889° W), was selected. This station is part of the CONAGUA monitoring network and provides a long, continuous record of daily precipitation data for extreme rainfall analysis.

It is important to emphasize that station 6001 is used solely for illustrative purposes in the presentation of results. The same data-processing workflow, exceedance analysis, and Generalized Extreme Value (GEV) modeling procedures were systematically applied to all meteorological stations included in the study.

### 2.2. Data Sources

The data used in this study primarily came from the National Water Commission’s Network of Meteorological Stations, which is available through the National Meteorological Service [7]. This network constitutes the primary in situ precipitation monitoring system in the region and provides long-term, sensor-based observations of daily rainfall.

**Figure 1:**
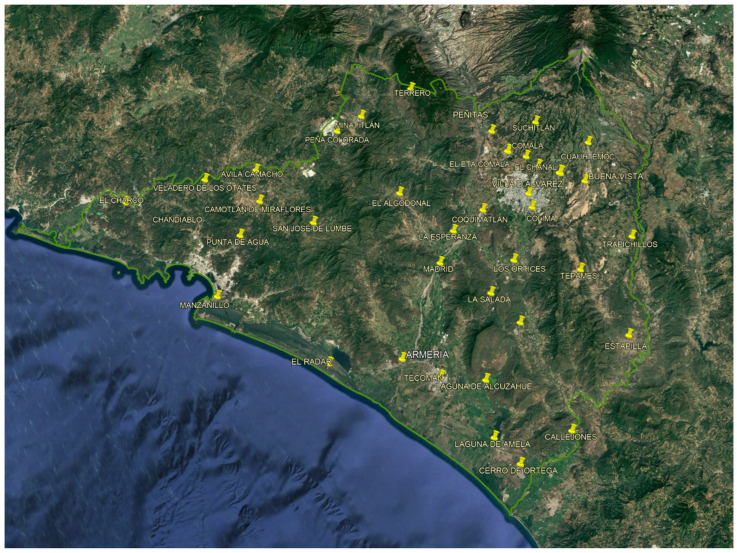
Stations CONAGUA in the state of Colima.

Daily precipitation records were collected from 37 weather stations located within the state of Colima and its border areas with Jalisco and Michoacán, as shown in Figure 1.

The stations were selected based on institutional data availability and record completeness, with the objective of achieving the most extensive spatial and temporal coverage possible. It is acknowledged that this selection strategy may introduce spatial sampling bias, particularly given the higher station density in coastal areas compared to mountainous regions. This limitation is explicitly considered in the interpretation of the spatial interpolation results.

The study period spanned 1980 to 2023, providing over 4 decades of historical data. The data were downloaded in CSV and TXT formats and standardized to a consistent field structure, including date, daily precipitation (in millimeters), station, latitude, longitude, and altitude. This harmonized dataset served as input for the exceedance analysis, extreme-value modeling, and spatial interpolation procedures, which were applied uniformly across all stations.

### 2.3. Exceedance Analysis

The exceedance analysis focused on identifying days when the daily precipitation recorded at each station was equal to or greater than 50 mm. This threshold was selected in accordance with the operational criteria of the National Center for Disaster Prevention (CENAPRED) and the World Meteorological Organization (WMO), which consider this value to be representative of intense rainfall capable of generating flash floods, urban flooding, or landslides [8].

Selecting a 50 mm threshold aligns with international studies that use similar criteria to identify extreme precipitation events, as this level typically marks the transition between localized, intense rainfall and episodes with the potential for severe hydrological impact [9,10].

For each meteorological station, the number of days with rainfall exceeding 50 mm per year was determined to estimate the annual exceedance frequency. The procedure was based on the formal definition of exceedance frequency proposed by Ben-Zvi [11] and adopted in various hydrological studies [12,13].

The mathematical expression is as follows:

$$E_{a}=\sum_{i=1}^{n_{a}}I(P_{i}\geq T)$$

where

$E_{a}$ = annual number of exceedances,
$n_{a}$ = total number of observations in year *a*,
I = indicator function (1 if $P_{i}\geq T$; 0 otherwise).

Subsequently, the multi-annual average of exceedances was calculated for each station:

$$E_{prom}=\frac{1}{m}\sum_{a=1}^{m}E_{a}$$

where *m* corresponds to the total number of years with available data.

The result represents the average annual frequency of intense events, facilitating the comparison of precipitation patterns across different regions of the state. The values obtained were organized into a database that served as input for statistical modeling of extreme values (GEV distribution) and subsequent spatial interpolation in Python (version 3.13) and QGIS (version 3.38).

In this way, the exceedance analysis constituted the starting point for the probabilistic and geospatial characterization of intense rainfall in Colima, in accordance with the methodology proposed by recent studies on the use of rainfall thresholds and extreme modeling [13].

### 2.4. Model GEV

Based on the annual series of maximum daily precipitation, the Generalized Extreme Value (GEV) distribution was fitted using the genextreme.fit() function from the scipy.stats (version 1.10) package in Python (version 3.13). This library allows robust estimation of the location (μ), scale (σ), and shape (ξ) parameters using the maximum likelihood method [14].

The estimated parameters describe the variability and asymmetry of annual maximum values, enabling characterization of the statistical behavior of extreme events. Once the parameters were determined, the return periods (T) were calculated using the following relationship:

$$T=\frac{1}{1-F(x)}$$

where F(x) is the cumulative distribution function (CDF) of the GEV distribution. This procedure allows estimating the magnitude of precipitation associated with different return periods—for example, 2, 5, 10, 25, 50, and 100 years—based on the quantiles of the fitted probability function.

For each meteorological station, return curves (precipitation vs. return period) were generated, graphically representing the relationship between the magnitude of the extreme event and its expected frequency of occurrence. This approach, widely applied in recent hydrological studies, facilitates the identification of spatial and temporal patterns of extreme rainfall, while also serving as input for risk analysis and infrastructure planning [13,15].

### 2.5. Spatial Interpolation

Using the average exceedance values and the GEV model results, spatial interpolations were performed using the griddata function of SciPy (version 1.10) in Python (version 3.13) and, subsequently, the Inverse Distance Weighting (IDW) method in QGIS (version 3.38). These surfaces enabled the representation of areas with higher and lower frequencies of intense rainfall and were exported in raster format for geographic and cartographic analysis [16].

### 2.6. Data Quality Control

To ensure reliable results, a quality control procedure was applied to address missing data, station failures, and spatial bias. Years with more than 10% missing values were removed from the exceedance and GEV analyses, while shorter gaps were left as missing to avoid altering the statistical behavior of extremes.

Stations with prolonged reporting interruptions were identified using metadata from the National Meteorological Service, and only years that met minimum completeness criteria were retained. This prevented the underestimation of extreme events caused by equipment failures or administrative data gaps.

Finally, spatial sampling bias was considered, given the uneven distribution of meteorological stations—denser in coastal areas and sparse in mountainous zones. To mitigate this limitation, interpolation results were interpreted cautiously in poorly monitored regions, and two methods (SciPy’s griddata and QGIS IDW) were used to cross-validate spatial patterns, thereby improving the robustness of the geospatial analysis of extreme rainfall.

**Figure 2:**
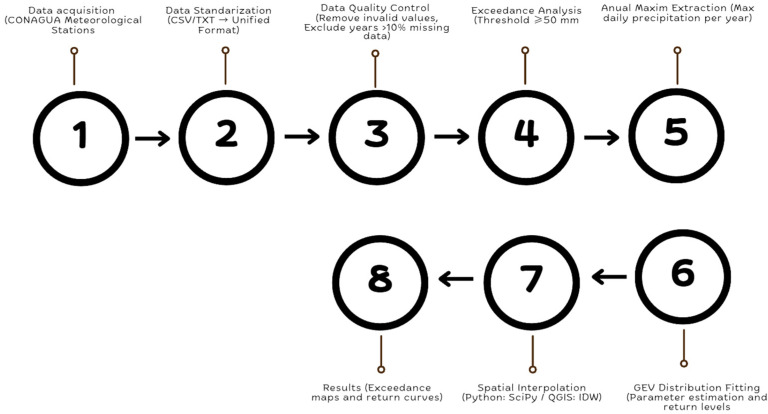
Methodological workflow for extreme rainfall analysis.

Figure 2 illustrates the methodological workflow applied in this study, from data acquisition and standardization to exceedance analysis, GEV modeling, and spatial interpolation, resulting in exceedance maps and return level curves.

## 3. Results

### 3.1. Analysis of Extreme Values

The exceedance probability of daily precipitation events (≥50 mm) was calculated following the procedure summarized in Algorithm 1. This approach ensures consistency and reproducibility across all analyzed meteorological stations.

The results indicate that for the analyzed period (1980–2023), station 6001 (Armeria, Colima) recorded a total of 26,225 valid days, of which 268 had rainfall equal to or greater than 50 mm, which corresponds to an exceedance proportion of 0.0102 (equivalent to 1.02% of the total observations).

This value suggests that, on average, the station records heavy rainfall on approximately 1 out of every 100 days in the historical record, confirming its occasional, but not permanent, exposure to high-magnitude events.

**Algorithm 1.** Exceedance analysis of daily precipitation (≥50 mm)Input:       Daily precipitation records P(d) for a given meteorological station       Threshold T = 50 mm Output:       Exceedance probability E Procedure:       1. Load daily precipitation data.       2. Convert precipitation values to numeric format.       3. Remove records with missing or invalid precipitation values.       4. Count total number of valid observations (N).       5. Count number of days where precipitation ≥ T (N_exceed).       6. Compute exceedance probability:                    E = N_exceed/N       7. Return E.

### 3.2. GEV Distribution

**Figure 3:**
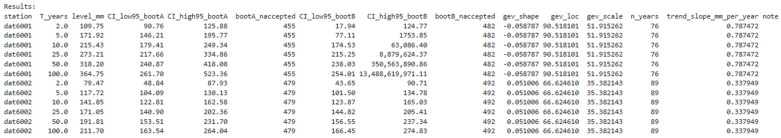
Output of the master file with the results of the GEV model applied to the stations in Colima.

Figure 3 presents a fragment of the master file of results obtained after applying the Generalized Extreme Value (GEV) model to the annual maximum precipitation series of the meteorological stations of the state of Colima.

The model was systematically applied to the 32 stations with complete and consistent historical records, selected based on their spatial and temporal coverage. The results were stored in CSV format, including the model’s statistical parameters and estimated maximum precipitation values for return periods (T) of 2, 5, 10, 25, 50, and 100 years.

Each record in the file includes the following fields:

- station: code for the weather station.
- T_years: years for each return period (2, 5, 10, 25, 50, 100)
- level_mm: max rainfall (mm) for each return period.
- CI_low95 and CI_high95: lower and upper 95% confidence intervals, from bootstrapping.
- gev_shape, gev_loc, gev_scale: GEV model’s shape, location, and scale.
- boot_naccepted: number of bootstrap samples used for confidence intervals.

**Figure 4:**
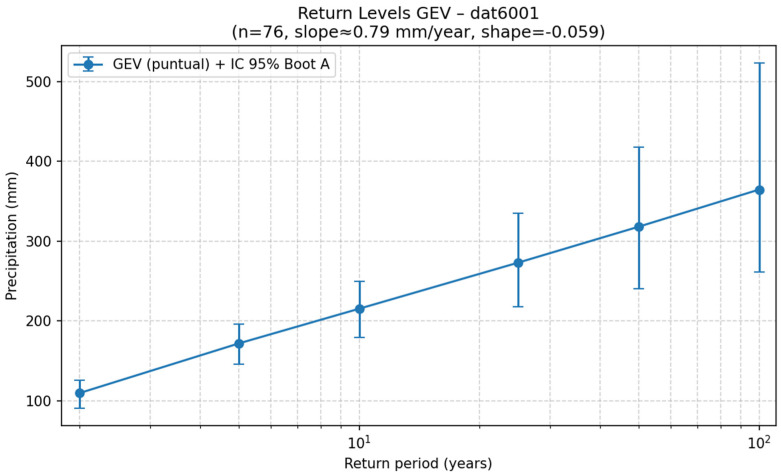
Return level curve obtained for station 6001 (Armeria, Colima).

The example shown in Figure 4 corresponds to station 6001 (Armeria, Colima), where a progressive increase in estimated precipitation is observed as the return period increases: for a 2-year return period, a maximum rainfall of 109.75 mm was estimated, for a 10-year return period, 215.43 mm, and for a 50-year return period, 318.20 mm.

This upward trend is consistent with the station’s observations and with the theoretical behavior of the GEV distribution, which models the probability of extreme events. This pattern confirms that higher-intensity events are less frequent but more severe, providing a solid basis for hydrometeorological risk management in the region.

The comprehensive analysis of the 37 stations yielded a set of return curves and the determination of the maximum expected rainfall for different time horizons.

These results were subsequently used to develop return period interpolation maps to identify areas of the state with the greatest potential to experience extreme rainfall, thereby strengthening the spatial interpretation of the risk associated with intense precipitation.

### 3.3. Return Level Curve GEV for Station

Figure 4 presents the return level curve obtained for station 6001 (Armeria, Colima), fitted using the Generalized Extreme Value (GEV) distribution.

The X-axis shows the return period (years) on a logarithmic scale, while the Y-axis represents the estimated maximum precipitation (mm) for each time interval. The points indicate the model’s estimated point values, and the vertical bars correspond to 95% confidence intervals calculated using the bootstrap method.

There is a progressive increase in expected precipitation as the return period increases, reflecting the natural tendency of extreme events: the longer the recurrence interval, the more severe the associated rainfall.

In this case, the GEV model estimated that, over a 2-year period, the maximum probable precipitation is approximately 110 mm; over a 50-year period, it could reach about 320 mm; and over a 100-year period, it could exceed 350 mm.

Taken together, this curve illustrates the GEV model’s ability to accurately represent the relationship between precipitation magnitude and frequency of occurrence, providing key information for sizing hydraulic works, land planning, and estimating flood risk.

**Figure 5:**
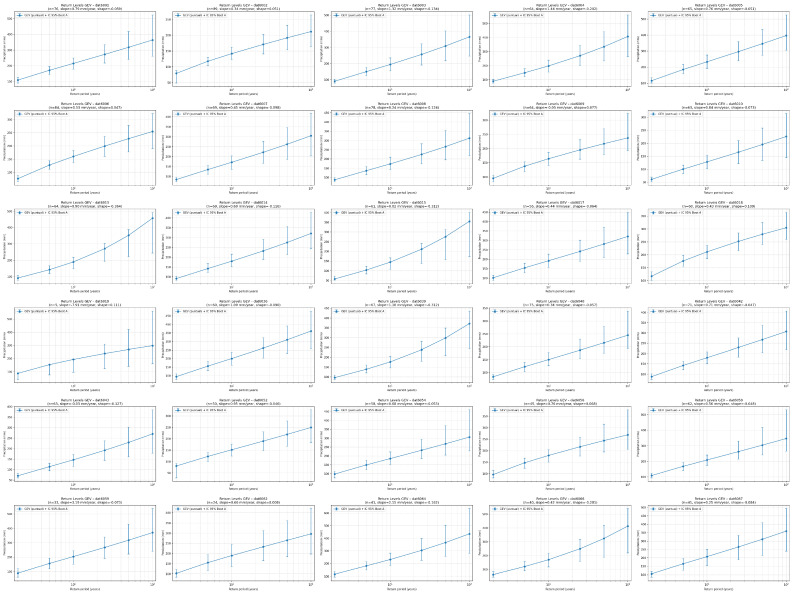
Return curves were generated for the 32 meteorological stations in the state of Colima.

Figure 5 presents the return curves generated for the 32 meteorological stations in the state of Colima, derived from the Generalized Extreme Value (GEV) model applied to the series of annual maximum daily precipitation values.

### 3.4. Spacial Interpolation

Analysis of daily precipitation records revealed marked spatial and temporal variability in the state of Colima. Annual rainfall values showed a strong concentration between June and October, coinciding with the tropical cyclone season in the Mexican Pacific.

The results of exceedance frequency ≥ 50 mm showed that the northern and western areas of the state—particularly the Minatitlán, Comala, and Peña Colorada stations recorded the highest values, with averages exceeding 20 days per year.

**Figure 6:**
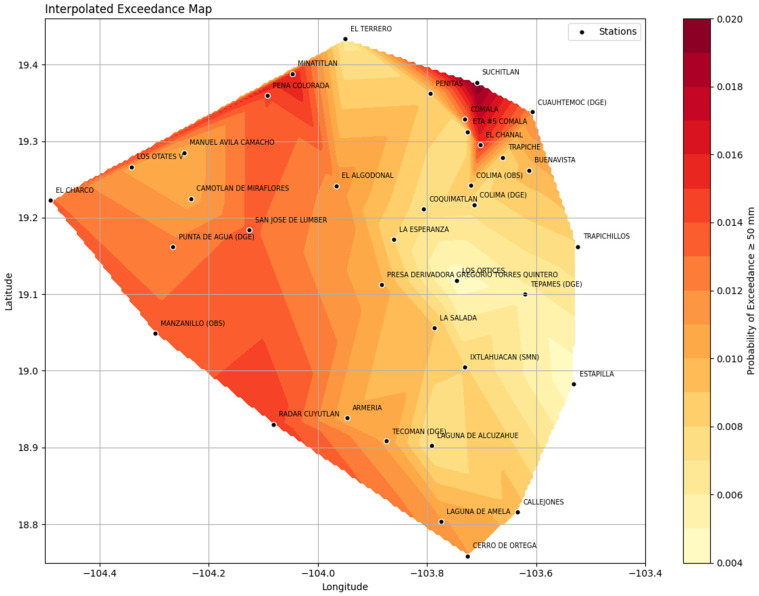
Spatial Interpolation with exceedance frequency of precipitation (≥50 mm), period 1980–2023.

In contrast, the southern and southeastern stations, such as Tecomán, Cerro de Ortega, and Laguna de Amela, exhibited less recurrence, with values ranging from 5 to 8 days per year, as shown in Figure 6.

The map shows the spatial distribution of the probability of exceeding 50 mm of rainfall at meteorological stations in the state of Colima. In other words, it represents the frequency of heavy rainfall across the state.

The darker colors (intense reds and oranges) indicate areas where the probability of rainfall exceeding 50 mm is higher, while yellow and light tones represent regions with a lower frequency of these events.

## 4. Discussion

This study demonstrates that combining exceedance analysis, GEV modeling, and spatial interpolation offers a robust tool for characterizing the extreme rainfall regime in Colima. The results successfully address the main objective of estimating the recurrence of high-intensity events and identifying their spatial distribution across the state. Together, these analyses strengthen the evidence base needed for hydrometeorological risk management under conditions of increasing climate variability.

The results demonstrated marked differences in rainfall intensity, primarily attributable to the presence of mountains in the state’s central and northern regions and to proximity to the Pacific Ocean along the western coastline. This distribution aligns with earlier research indicating more intense extreme weather in the mountain ranges of western Mexico [17,18]. Additionally, the estimated rainfall map corroborates this pattern, showing a high likelihood of heavy rainfall in the northern part of the state, while the western coastal regions experience more consistent rainfall year-round. These spatial gradients are further shaped by the uneven distribution of meteorological stations, which are more concentrated along the coast and sparser in the elevated, mountainous inland regions.

The use of the GEV distribution offers substantial advantages over other statistical approaches—such as Gaussian or empirical regression models—because it focuses on the upper tail, where extreme values are concentrated. This enabled a more accurate estimate of the probability of low-frequency, high-magnitude rainfall events, which is essential for the design of hydraulic infrastructure and urban planning.

Integrating grid data (SciPy) and IDW (QGIS) methods for interpolation produced detailed spatial results. This approach clearly identifies high-probability exceedance zones. These tools improve territorial analysis and support global guidelines to use open-source technologies in climate risk management [4,19]. However, interpolation becomes less precise where station density is low, so interpret rasters in these areas carefully.

The methodological results underscore the importance of consistent, continuous historical data series. These data are crucial for reliably fitting probabilistic models. However, western Mexico still faces a significant challenge due to the low density of meteorological stations and missing records in some years.

These limitations highlight the importance of incorporating additional data sources—such as satellite-based precipitation estimates and higher-resolution ground sensors—to improve spatial coverage and temporal resolution. Artificial intelligence methods, especially U-Net or LSTM architectures, may also help refine downscaling and pattern-recognition processes to enhance flood monitoring capabilities.

## 5. Conclusions

The study established a replicable methodological framework for the probabilistic and spatial assessment of extreme rainfall in regions with limited monitoring infrastructure. The combination of exceedance analysis, GEV distribution, and spatial interpolation techniques yielded consistent, visually interpretable results, which are essential for informed decision-making in water risk management.

The proposed approach not only enhances understanding of historical precipitation patterns in Colima but also lays the groundwork for developing hybrid predictive models. The future integration of artificial intelligence, IoT sensors, and satellite imagery will enable progress toward automated early warning systems capable of anticipating and mitigating the impacts of extreme hydrometeorological events.

This type of tool represents a decisive step toward adaptive and resilient water management in the context of climate change, consolidating the transition from a reactive to a proactive approach in the state of Colima’s territorial planning.

## Figures and Tables

**Table 1:** Hydrometeorological phenomena have affected the state of Colima.

Type	Place	Date
Declaration of natural disaster (hurricane, severe rain, river, and pluvial flooding).	In seven municipalities of the State of Colima, Manzanillo is included.	28–29 August 2021
Declaration of emergency due to severe rainfall and fluvial flooding.	Municipality of Manzanillo, Colima.	26–27 August 2020
Declaration of emergency due to severe rain, river flooding, and pluvial flooding.	In ten municipalities of the state of Colima.	29–30 September 2019
Declaration of Natural Disaster (severe rain and pluvial and fluvial flooding).	In four municipalities of the state of Colima.	18–19 September 2019
Declaration of extraordinary emergency (imminence and high probability of impact of hurricane “Patricia.”)	In ten municipalities of the state of Colima.	30 October 2015
Emergency declaration of tropical storm “Manuel” (severe rain).	In seven municipalities of the state of Colima.	15–17 September 2013
Declaration of emergency due to the presence of hurricane “JOVA.”	In ten municipalities of the state of Colima.	12 October 2011

## Data Availability

The data used in this study are publicly available from the National Meteorological Service (CONAGUA) of Mexico.

## Abbreviations

The following abbreviations are used in this manuscript:

IoT	Internet of Things
AI	Artificial Intelligence
UNDRR	United Nations Office for Disaster Risk Reduction
WMO	World Meteorological Organization

## References

[1] (2021). Sequías, Tormentas e Inundaciones: El Agua y el Cambio Climático Dominan la Lista de Desastres. https://onu-habitat.org/index.php/sequias-tormentas-e-inundaciones-el-agua-y-el-cambio-climatico-dominan-la-lista-de-desastres.

[2] Centro Nacional de Prevención de Desastres (CENAPRED) (2024). Resumen Ejecutivo del Impacto de los Principales Desastres Ocurridos en México Durante 2023. Secretaría de Seguridad y Protección Ciudadana. https://www.cenapred.gob.mx/es/Publicaciones/archivos/504-RESUMENEJECUTIVOIMPACTO2023.PDF.

[3] United Nations Office for Disaster Risk Reduction (UNDRR) (2024). Snapshot of Sendai Framework Monitoring—Number of Disaster-Affected People per 100,000 Population, 2005–2014 to 2014–2023. https://www.undrr.org/monitoring-sendai-framework/snapshot.

[4] Sendai Framework for Disaster Risk Reduction 2015–2030. https://www.undrr.org/publication/sendai-framework-disaster-risk-reduction-2015-2030.

[5] World Meteorological Organization (WMO) (2024). State of the Global Climate 2023.

[6] Held I.M., Soden B.J. (2006). Robust Responses of the Hydrological Cycle to Global Warming. J. Clim..

[7] Comisión Nacional del Agua (CONAGUA) (2024). Normales Climatológicas por Estado: Red de Estaciones Meteorológicas. Servicio Meteorológico Nacional. https://smn.conagua.gob.mx/es/climatologia/informacion-climatologica/normales-climatologicas-por-estado.

[8] CENAPRED (2020). Resumen Ejecutivo del Impacto de los Principales Desastres Ocurridos en México Durante 2020.

[9] Ramos Filho G.M., França G.B., Gomes L.O. (2021). An improved rainfall-threshold approach for robust prediction and warning of flood and flash flood hazards. Nat. Hazards.

[10] Burszta-Adamiak E., Licznar P., Łomotowski J. (2016). Criteria for identifying maximum rainfall determined by the partial duration series approach. Meteorol. Hydrol. Water Manag..

[11] Ben-Zvi A. (2009). Rainfall intensity–duration–frequency relationships derived from large partial duration series. J. Hydrol..

[12] Kundzewicz Z.W., Kanae S., Seneviratne S.I., Handmer J., Nicholls N., Peduzzi P., Mechler R., Bouwer L.M., Arnell N., Mach K. (2014). Flood risk and climate change: Global and regional perspectives. Hydrol. Sci. J..

[13] Pan X., Rahman A., Azhari F., Bhuiyan M.A.H. (2022). Peaks-over-threshold model in flood frequency analysis: A scoping review. Stoch. Environ. Res. Risk Assess..

[14] SciPy Documentation scipy.stats.genextreme—SciPy v1.16.2 Manual. https://docs.scipy.org/doc/scipy/reference/generated/scipy.stats.genextreme.html.

[15] Aengenheyster M., Reinders J. Tutorial 4: Return Levels Using Normal and GEV Distributions. Climate Match Academy. https://comptools.climatematch.io/tutorials/W2D3_ExtremesandVariability/student/W2D3_Tutorial4.html.

[16] QGIS Development Team (2024). Interpolation—QGIS Documentation (Version 3.40). Open Source Geospatial Foundation Project. https://docs.qgis.org/latest/en/docs/user_manual/processing_algs/qgis/interpolation.html.

[17] Rincón-Ávalos P., Bravo-Cabrera J.L., Hernández-Santana J.R. (2022). Rainfall extremes and climate variability in western Mexico: Trends and spatial patterns derived from observational datasets. Atmósfera.

[18] Mendoza-Cano O., López de la Cruz J., Pattison I., Martínez-Preciado M., Uribe-Ramos J.M., Edwards R.M., Ramírez-Lomelí C.I., Rincón-Ávalos P., Velazco-Cruz J.A. (2021). Disaster Risk Resilience in Colima–Villa de Álvarez, Mexico: Application of the Resilience Index to Flash Flooding Events.

[19] World Meteorological Organization (WMO) (2023). State of the Climate in Latin America and the Caribbean 2023.

